# Fear, Psychological Impact, and Coping During the Initial Phase of COVID-19 Pandemic Among the General Population in India

**DOI:** 10.7759/cureus.20317

**Published:** 2021-12-10

**Authors:** Gautami Nagabhirava, Sangha Mitra Godi, Akhil D Goel

**Affiliations:** 1 Psychiatry, Kamineni Academy of Medical Sciences and Research, Hyderabad, IND; 2 Psychiatry, All India Institute of Medical Sciences, Raipur, IND; 3 Community and Family Medicine, All India Institute of Medical Sciences, Jodhpur, IND

**Keywords:** coronavirus, stress, depression, anxiety, fear, mental health, pandemic, covid-19

## Abstract

Introduction

Public health emergencies such as pandemics affect the health, safety, and well-being of both individuals and societies. Thus, this study aims to better understand the fear due to coronavirus disease (COVID) and associated levels of anxiety, depression, stress, and coping in the general public of India during the initial stage of the COVID-19 outbreak.

Materials and methods

This was a cross-sectional study to assess the psychological impact of COVID-19 and coping levels among the general population during the coronavirus pandemic’s initial phase. An online survey was conducted using a snowball sampling technique.

Results

A total of 489 people responded to the survey. The prevalence rates of depression, anxiety and stress were 27.2%, 21.5%, and 15.3% respectively. Female gender, age below 35 years, history of medical or psychiatric illness, and those who had personal contact with persons with COVID-19 were significantly associated with presence of depression, anxiety, and stress whereas spending more than 1 hour on COVID-19-related information was associated with significant stress.

Conclusion

This study concludes that the prevalence rates of psychological problems were high during the COVID-19 pandemic. These are directly related to the fear associated with COVID-19 but had an inverse relationship with the resilient coping levels.

## Introduction

Public health emergencies such as pandemics affect individuals’ and societies’ health, safety, and well-being. Hence, they affect not only physical health but also mental health intimately due to their unprecedented nature [1,2]. The World Health Organization (WHO) declared the coronavirus disease 2019 (COVID-19) outbreak a global pandemic on March 11, 2020 [3]. The Indian government also acted swiftly, and the largest national lockdown in the world was imposed on 25th March for 21 days which was later extended to May 3, 2020, in response to the pandemic. Although the WHO praised this response as “tough and timely,”[4] the coronavirus itself has plunged the world into uncertainty and induced fear in the general public with people worrying about themselves or their loved ones contracting the virus. Mandatory contact tracing and restricted movement as a public health response also further contribute to the general population’s increased anxiety, guilt, and stigma [5].

Though lockdown is necessary to curb community spread and break the infection cycle, it can also affect people’s psychological health and generate much turmoil [6]. It is an unpleasant and unfamiliar experience with separation from loved ones, loneliness, loss of freedom, and boredom at times, causing a high prevalence of psychological symptoms of distress and disorder [7]. Previous research during the severe acute respiratory syndrome (SARS) epidemics has shown that poor coping mechanisms, a high degree of anxiety, pre-existing illness, and unverified information were risk factors in developing psychological problems [8,9].

In a recent meta-analysis, the prevalence rates of stress, anxiety, and depression due to the COVID-19 pandemic were 29.6, 31.9, and 33.7%, respectively, in the general population [10]. The prevalence rates of anxiety among the general population were also found to be more than three times higher during the COVID-19 pandemic compared to normal times [11]. Thus, this study aims to better understand the fear due to COVID and associated levels of anxiety, depression, stress, and coping in the general public of India during the initial stage of the COVID-19 outbreak.

## Materials and methods

This was a cross-sectional study to assess the general public’s psychological response during the initial phase of the coronavirus pandemic, particularly the impact of lockdown and the beginning of the quarantine (April 12 to April 18, 2020). A snowball sampling technique was utilized to recruit the general public in India, and it was first distributed to college students through email and WhatsApp who were then asked to circulate it to others. The respondents completed the survey using an online survey platform (Google forms). Post-completion, participants with severe scores were advised to seek help along with listed mental health resources. Institutional ethics committee approval was obtained from Kamineni Hospitals on April 7, 2020 (Registration #: ECR/58/Inst/AP/2013/RR-19).

Sociodemographic details were collected such as age, sex, education, residential location, current living status, employment status, and medical/psychiatric illness. Respondents were also asked questions regarding time spent on coronavirus news (discussing/watching), source of information, contact history, and perceived risk of contracting the coronavirus.

The psychological impact of coronavirus was measured using the Depression, Anxiety, Stress Scale-21 (DASS-21), which is an easy-to-use instrument that has been used in research related to both COVID-19 as well as other infectious disease outbreaks multiple times [11-12]. The questionnaire was divided into three subscales: depression; anxiety; and stress. The total depression score was divided into normal (0-9), mild depression (10-13), moderate depression (14-20), severe depression (21-27), and extremely severe depression (28-42). The anxiety subscale score was divided into normal (0-7), mild anxiety (8-9), moderate anxiety (10-14), severe anxiety (15-19), and extremely severe anxiety (20-42). The stress subscale score was divided into normal (0-14), mild stress(15-18), moderate stress (19-25), severe stress (26-33), and extremely severe stress (34-42). This questionnaire has been provided in the Appendices Section.

The fear of coronavirus disease-19 scale (FCoV-19s) consists of seven items and measures the fear of coronavirus disease with each statement on a 5-point scale from 1-Strongly disagree to 5-Strongly agree with subjects rating their agreement [13].

Coping and resilience were assessed using the 4-item Brief Resilient Coping Scale (BRCS). The scale was divided into low-resilient copers (4-13), medium-resilient copers (14-16 points), and high-resilient copers (17-20) [8].

The data collected was analyzed using SPSS 23.0 version (SPSS Inc., Chicago, IL, USA). Descriptive statistics were calculated for sociodemographic data and the study population’s prevalence of depression, anxiety, and stress levels. The chi-square test was used to find the association for categorical variables, and correlation statistics were used for continuous variables to find the strength of association. A p-value of less than 0.05 was considered statistically significant.

## Results

During the study, a total of 489 responses were collected from 21 states in India. The sociodemographic profile of the sample revealed that 51.3 % of the subjects were males while the age distribution of respondents for 18-24 years was 31.5%, 25-35 years was 39.9%, 36-45 years was 10%, 45-60 years was 12.7%, and >60 years was 5.9%. About 60.9% were from non-medical backgrounds, 49.9% were single, but 82.6% lived with the family during the lockdown period. The study found that 33.6% of the subjects worked as part of the essential COVID-19 services during the lockdown, and 42.5% reported working from home. The primary source of information regarding COVID-19 during lockdown was news and social media in 62.2 % and 14.7% of the sample, respectively. During the lockdown, 41.9% of the individuals reported spending more than 1 hour on average on COVID-19 discussion/news/statistics, with 6.7% spending more than 4 hours. About 44.4% of the subjects reported their perceived level of risk for contracting the COVID-19 as low; however, 47.3% were extremely concerned that a family member might contract the COVID-19.

The psychological impact was assessed by using the DASS-21. Table 1 shows the prevalence rates of depression, anxiety, and stress with the levels of severity. The prevalence of depression, anxiety, and stress was found to be 27.2%, 21.5%, and 15.3% respectively. A total of 8.2% were considered to suffer from mild depression, 9.8% from moderate depression, 3.9% from severe, and the remaining 5.3% from extremely severe depression. Of the total sample, 3.7% were considered to suffer from mild anxiety, 9.0% from moderate anxiety, 3.1% from severe, and 5.7% from extremely severe anxiety. About 4.7% were considered to suffer from mild stress, 3.7% from moderate stress, 7% from severe to extremely severe stress levels. Resilience and coping were measured through the BRCS. About 39.1% of the respondents were low resilient copers while 37.8% and 23.1%, respectively, were medium- and high-resilient copers as shown in Table 1.

**Table 1:** Prevalence of Depression, Anxiety, Stress, and Resilient Coping

Parameter	n (489)	%
Depression screening		
Score ≤ 9 (Not depressed)	356	72.8
Mild (10-13)	40	8.2
Moderate (14-20)	48	9.8
Severe (21-28)	19	3.9
Extremely severe (>28)	26	5.3
Anxiety screening		
Score ≤7 (Not anxious)	384	78.5
Mild (8-9)	18	3.7
Moderate (10-14)	44	9.0
Severe (15-19)	15	3.1
Extremely severe (>20)	28	5.7
Stress screening		
Score ≤14 (Not stressed )	414	84.7
Mild (15-18)	23	4.7
Moderate (19-25)	18	3.7
Severe (26-33)	21	4.3
Extremely severe (>34)	13	2.7
Coping		
Low resilient (4-13)	191	39.1
Medium resilient (14-16)	185	37.8
High resilient (17-20)	113	23.1
(Total number of participants=489)		

 

Table 2 shows the association of depression, anxiety, and stress levels with various social and demographic factors. It was found that female gender, age less than 35 years, history of medical or psychiatric illness, and those who had personal contact with persons with COVID-19 were significantly associated with the presence of depression, anxiety, and stress levels. Being single was significantly associated with stress and depression, whereas substance use and spending more than 1 hour on COVID-19-related information/news was associated with significant stress.

**Table 2:** Association of Depression, Anxiety, and Stress as per DASS-21 in Relation To Various Sociodemographic Parameters DASS-21: Depression, Anxiety, Stress Scale-21

		Stress					Depression					Anxiety				
		Yes		No		p-value	Yes		No		p-value	Yes		No		p-value
Gender	Male	23	30.7%	228	55.1%	<0.001	55	41.4%	196	55.1%	0.007	39	37.1%	212	55.2%	<0.001
	Female	52	69.3%	186	44.9%		78	58.6%	160	44.9%		66	62.9%	172	44.8%	
Age	<35 years	64	85.3%	285	68.8%	0.003	105	78.9%	244	68.5%	0.023	85	81.0%	264	68.8%	0.014
	35 years and above	11	14.7%	129	31.2%		28	21.1%	112	31.5%		20	19.0%	120	31.3%	
Marital status	Single	46	61.3%	198	47.8%	0.031	77	57.9%	167	46.9%	0.031	59	56.2%	185	48.2%	0.146
	Married	29	38.7%	216	52.2%		56	42.1%	189	53.1%		46	43.8%	199	51.8%	
Lockdown status	Living Alone—Solitary	13	17.3%	72	17.4%	0.99	27	20.3%	58	16.3%	0.298	16	15.2%	69	18.0%	0.513
	Living with family/ Significant other	62	82.7%	342	82.6%		106	79.7%	298	83.7%		89	84.8%	315	82.0%	
work/study from home	Yes	39	52.0%	169	40.8%	0.072	61	45.9%	147	41.3%	0.363	46	43.8%	162	42.2%	0.766
	No	36	48.0%	245	59.2%		72	54.1%	209	58.7%		59	56.2%	222	57.8%	
Essential services	Yes	18	24.0%	145	35.0%	0.062	42	31.6%	121	34.0%	0.615	38	36.2%	125	32.6%	0.483
	No	57	76.0%	269	65.0%		91	68.4%	235	66.0%		67	63.8%	259	67.4%	
Past medical/psychiatric history	Yes	28	37.3%	56	13.5%	<0.001	45	33.8%	39	11.0%	<0.001	32	30.5%	52	13.5%	<0.001
	No	47	62.7%	358	86.5%		88	66.2%	317	89.0%		73	69.5%	332	86.5%	
Substance use	Yes	20	26.7%	72	17.4%	0.059	28	21.1%	64	18.0%	0.439	26	24.8%	66	17.2%	0.078
	No	55	73.3%	342	82.6%		105	78.9%	292	82.0%		79	75.2%	318	82.8%	
Time spent	<1 hour	35	46.7%	249	60.1%	0.03	70	52.6%	214	60.1%	0.136	55	52.4%	229	59.6%	0.182
	1 hour and above	40	53.3%	165	39.9%		63	47.4%	142	39.9%		50	47.6%	155	40.4%	
Personal contact	Yes	11	14.7%	26	6.3%	0.012	16	12.0%	21	5.9%	0.023	13	12.4%	24	6.3%	0.035
	No	64	85.3%	388	93.7%		117	88.0%	335	94.1%		92	87.6%	360	93.8%	
Perceived level of risk	Low	35	46.7%	182	44.0%	0.173	53	39.8%	164	46.1%	0.358	41	39.0%	176	45.8%	0.463
	Medium	29	38.7%	132	31.9%		50	37.6%	111	31.2%		38	36.2%	123	32.0%	
	High	11	14.7%	100	24.2%		30	22.6%	81	22.8%		26	24.8%	85	22.1%	

**Figure 1:**
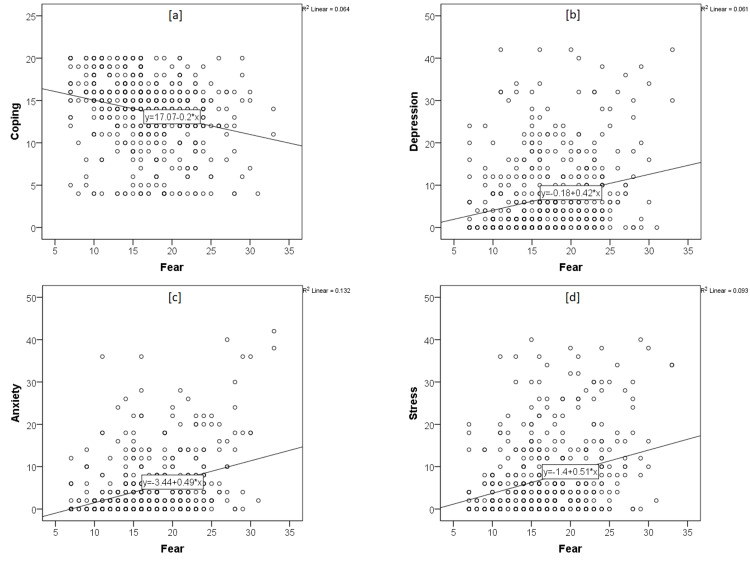
The scatter plots of correlation statistics between DASS-21, FCoV-19S, and BRCS scores DASS: Depression, Anxiety, Stress Scale; FCoV-19: Fear of COVID-19 Scale; BRCS: Brief Resilient Coping Scale

The scatter plot (Figure 1) shows the correlation statistics, and there was a positive correlation between scores of FCoV-19s and the scores of DASS-21 subscales of anxiety (r=0.364, p<0.01), depression (r=0.247, p<0.01), and stress(r=0.306, p<0.01). Meanwhile, the scores of BCRS had a negative correlation with scores of FCoV-19s (r=−0.253, p<0.01), as well as depression (r=−0.107, p<0.05), anxiety (r=−0.102, p<0.05), and stress (r=−0.91, p<0.05) subscales of DASS-21. There was also a significant positive correlation between depression and anxiety (r=−0.773, p<0.01) and stress subscale scores (r=0.859, p<0.01) (Table 3).

**Table 3:** Pearson’s Correlation of the Fear, Coping, and DASS-21 **Correlation is significant at 0.01 level (two-tailed). *Correlation is significant at 0.05 level (two-tailed).

	Fear	Coping	Depression	Anxiety	Stress
Coping	-0.253^**^	1	-0.107^*^	-0.102^*^	-0.091^*^

## Discussion

Our research findings are consistent with other studies in the literature showing that COVID-19 has a significant impact on mental health. The prevalence of depression, anxiety, and stress was 27.2%, 21.5%, and 15.3%, respectively, and this is almost similar but relatively low compared to a recent meta-analysis by Salari et al. (2020) and a cross-sectional survey by Turna et al. (2021) of 632 individuals [10,14]. The prevalence rates of anxiety, depression, and stress levels during the COVID-19 pandemic in the general population sample of other countries also showed similar rates [15-19].

The female gender was significantly associated with stress, depression, and anxiety (p<0.05). This is similar to studies worldwide where gender differences have been noted with an increased biological sensitivity of women to stress [18,20,21]. Moreover, most women faced the responsibility of being the family’s main caregiver with the added burden of child-rearing, domestic work without additional help during the lockdown, making them more vulnerable to higher levels of anxiety and depression [17]. The higher FCoV-19s scores in females than males in the current study might have contributed to the high psychological impact of COVID-19 in females.

Age less than 35 years was also significantly associated with stress, anxiety, and depression consistent with previous studies. This could be attributed to a multitude of reasons, including uncertainty regarding future and career, boredom and frustration due to lockdown, and increased exposure to social media during the pandemic. Additional history of previous medical and psychiatric illness was also significantly associated with stress, anxiety, and depression. This echoes current literature and has been explained by the possibility that those with pre-existing conditions are at increased risk for psychological distress [16,22,23]. Moreover, these individuals also seem to evaluate themselves as more vulnerable to any new infections [21]. Those employed in essential services had lower DASS item scores than the rest of the study population. One possible explanation could be the absolute necessity and utility of their services to maintain health and welfare, leading to quick adaptation to the pandemic. Moreover, feeling useful and being occupied with work may also distract from pandemic-related negative thoughts and worries. This is also explained by the significantly higher mean scores of resilient coping in people working in essential services than non-essential services. Interestingly, lockdown status and working from home independently did not have any bearing on the DASS items scores. In the current study, married people had significantly lower scores of stress and depression, suggesting that marriage might act as a buffer and additional support to help combat stress.

The finding that the time spent over information about COVID-19 for more than 1 hour was associated with significant stress, anxiety, and depression is consistent with previous studies [17,18] as the excess time spent results in excess and/or repetitive exposure to misinformation and online health or COVID-19-related health search. Increased usage of online searches for medical information and the ensuing anxiety known as cyberchondria is considered as an independent risk factor for increased anxiety levels in the setting of COVID-19 [24-26].

The mean score of FCoV-19s was 16.8 (SD=5.27), which was slightly lower than other studies such as Doshi et al.’s [20] and another study by Giordani et al. [27], which reported 18.00 (SD=5.68) and 19.8 (SD=5.3), respectively. The lower score could be attributed to the difference in the pandemic stages, with India still in its initial phase. There was a significant positive correlation between depression and anxiety (ρ=−0.773, p<0.01) and stress (ρ=0.859, p<0.01). This could be due to similar pathophysiology and similarity of the items in the questionnaire. Furthermore, both anxiety and depression are often comorbid and interrelated to ongoing stress levels and the degree of strength of the stressor.

Finally, our results indicated that higher coping and resilience scores were associated with reduced FCoV-19s and DASS scale scores. This finding helps corroborate existing literature’s evidence that coping plays a vital role in protecting and improving mental health in individuals facing health-related stress [28-30]. However, only 23.1% of our population met the criteria for high resilience copers. In comparison, 39.1 % and 37.8 had low- and medium-resilient coping, resulting in relatively higher rates of stress, anxiety, and depression in the current study.

There were limitations to the study. First, the study was conducted online and was a cross-sectional study. The small sample size and snowball sampling technique employed meant that the sample size could not be generalized to the general population and there was a risk of low external validity. The psychiatric diagnosis was based on screening instruments rather than a clinical diagnostic interview. Unfortunately, due to the study design, retesting and follow-up are not possible. A longitudinal study is warranted to understand the long-term effects of the pandemic.

## Conclusions

Our findings suggest that the COVID-19 pandemic and ensuing lockdown have led to a significant increase in psychological problems in the Indian general population. The Indian population must be granted access to timely mental health care, including psychotherapy and medication. Moreover, the research suggests that most of the study population has inadequate (low to medium) coping skills; hence, it is essential that we implement community-based strategies focusing on enhancing coping and caretaking behaviors to increase resilience. Proper implementation and access to telepsychiatry, an underutilized tool, can help individuals avail mental health services without the fear associated with in-person consultation during this COVID-19 crisis.

## Appendices

**Table 4:** Semi-structured Questionnaire: DASS-21, FCOV 19, and BRCS scales used for the study BRCS- Brief Resilient Coping Scale^8^ DASS-21 - Depression and Anxiety Stress Scale^12^ FCoV 19- Fear of COVID 19 Scale^13^

Q. No	Item		Response ✓										
1	Gender		Male										
			Female										
2	Age		18-24 years										
			25-35 years										
			36-45 years										
			46-60 years										
			>60 years										
3	City (Residence at present)												
4	Education												
5	Marital status		Single										
			Married										
			Widow/Widower										
6	What is your current living status in this period of lockdown?		Living alone										
			Living with significant other										
7	Profession												
8	Are you working at present in any of the following essential COVID-19 services?		Healthcare										
			Police										
			Sanitization services										
			Government public administrative services										
			Banking, electricity, postal department										
			Other										
			No, not working										
9	Do you need to work from home or attend classes online (students) during lockdown period?		Yes										
			No										
10	Medical Illness/Psychiatric illness? If yes, specify		Yes										
			No										
			Specify, if yes										
11	Do you use/ consume alcohol/tobacco/cannabis?		Yes										
			No										
12	What is your source of information regarding COVID-19?		News										
			Social media										
			Journals/publications										
			Friends/relatives/neighbors										
			Healthcare professionals										
			Other										
13	How much time do you spend on average on coronavirus news/statistics? (Watching/Reading/Forwarding/Discussing)		<1 hour										
			1-3 hours										
			4-7 hours										
			> 7 hours										
14	Do you personally know anyone who has been infected by the coronavirus?		Yes										
			No										
15	How do you rate your level of risk for contracting (catching) the coronavirus (COVID-19)?		High										
			Medium										
			Low										
16	How concerned are you that a family member will contract the coronavirus ?		Extremely concerned										
			Somewhat concerned										
			Not concerned at all										
	Rate your agreement from (1)- Strongly disagree to (5) -Strongly agree												
17	I am most afraid of COVID-19		1		2		3		4		5		
18	It makes me uncomfortable to think about COVID-19		1		2		3		4		5		
19	My hands become clammy when I think about COVID-19		1		2		3		4		5		
20	I am afraid of losing my life because of COVID-19		1		2		3		4		5		
21	When watching news and stories about COVID-19 on social media, I become nervous or anxious		1		2		3		4		5		
22	I cannot sleep because I'm worrying about getting COVID-19		1		2		3		4		5		
23	My heart races or palpitates when I think about getting COVID-19		1		2		3		4		5		
	Rate your agreement from (0)- Did not apply to me at all, (1)- Applied to me to some degree, (2)- Applied to me to a considerable degree, (3)- Applied to me very much												
24	I found it hard to wind down		0			1		2		3			
25	I was aware of dryness of mouth		0			1		2		3			
26	I couldn't seem to experience any positive feeling at all		0			1		2		3			
27	I experienced breathing difficulty (especially rapid breathing, breathlessness in the absence of physical exertion)		0			1		2		3			
28	I found it difficult to work up the initiative to do things		0			1		2		3			
29	I tended to over-react to situations		0			1		2		3			
30	I experienced trembling (e.g., in the hands)		0			1		2		3			
31	I was worried about situations in which I might panic and make a fool of myself		0			1		2		3			
32	I felt that I was using a lot of nervous energy		0			1		2		3			
33	I felt that I had nothing to look forward to		0			1		2		3			
34	I found myself getting agitated		0			1		2		3			
35	I found it difficult to relax		0			1		2		3			
36	I felt down-hearted and blue		0			1		2		3			
37	I felt intolerant of anything that kept me from getting on with what I was doing		0			1		2		3			
38	I felt I was close to panic		0			1		2		3			
39	I was unable to become enthusiastic about anything		0			1		2		3			
40	I felt I wasn't worth much as a person		0			1		2		3			
41	I felt that I was rather touchy		0			1		2		3			
42	I was aware of the action of my heart in the absence of physical exertion (sense of heart rate increase, heart missing a beat)		0			1		2		3			
43	I felt scared without any good reason		0			1		2		3			
44	I felt that life was meaningless		0			1		2		3			
	Rate your agreement from (1)-Does not describe me, (2)- Does not describe me, (3)- Neutral, (4)- Describes me, (5)- Describes me very well.												
45	I look for creative ways to alter difficult situations	1		2			3		4			5	
46	Regardless of what happens to me, I believe I can control my reaction to it	1		2			3		4			5	
47	I believe I can grow in positive ways by dealing with difficult situations	1		2			3		4			5	
48	I actively look for ways to replace the losses I encounter in life	1		2			3		4			5	
